# ﻿Phylogenetic evidence suggests the non-validity of the Iberian land snail genus *Tartessiberus* and confirms its synonymy with *Iberus* (Helicidae)

**DOI:** 10.3897/zookeys.1201.117318

**Published:** 2024-05-14

**Authors:** Michael J. Jowers, José Liétor, Antonio R. Tudela, Pedro A. Jódar, Inés Galán-Luque, Gregorio Moreno-Rueda

**Affiliations:** 1 CIBIO/InBIO (Centro de Investigação em Biodiversidade e Recursos Genéticos), Universidade do Porto, Campus Agrario De Vairão, 4485-661, Vairão, Portugal University of Granada Granada Spain; 2 BIOPOLIS Program in Genomics, Biodiversity and Land Planning, CIBIO, Campus de Vairão, 4485 661 Vairão, Portugal Universidade do Porto Vairão Portugal; 3 Departamento de Zoología, Facultad de Ciencias, Avenida de la Fuente Nueva s/n, 18071, University of Granada, Granada, Spain BIOPOLIS Program in Genomics, Biodiversity and Land Planning, CIBIO Vairão Portugal; 4 Departamento de Biología Animal, Biología Vegetal y Ecología, Campus Lagunillas s/n, 23071, University of Jaen, Jaen, Spain University of Jaen Jaen Spain; 5 Sociedad Giennense de Historia Natural, Capitán Aranda Baja, 12, 23001, Jaén, Spain Sociedad Giennense de Historia Natural Jaén Spain; 6 Sociedad Ibérica para el Estudio y Conservación de los Ecosistemas, Pol. Industrial Los Jarales, C/ Mina Alcolea s/n, 23700, Linares, Jaén, Spain Sociedad Ibérica para el Estudio y Conservación de los Ecosistemas Jaén Spain

**Keywords:** Andalusia, Gastropoda, Helicidae, Iberian Peninsula, land snails, morphometrics, new combination, Spain, *Tartessiberus*, taxonomy

## Abstract

The monospecific genus *Tartessiberus* was described in the year 2021 including a single species (*T.cilbanus*). However, its description relied solely on morphological and anatomical data. In the present work, we use a fraction of the mitochondrial DNA cytochrome oxidase subunit I (COI), 16S ribosomal RNA (16S rRNA) and the nuclear large ribosomal subunit (LSU) to clarify its validity through phylogenetic positioning. Knowledge of the distribution of this species is also improved by citing new locations and expanding the geographical range to approximately 200 km^2^. Additionally, a morphometric analysis of 259 shells is presented for comparisons with shells of the *Iberusmarmoratus* complex and testing the power of conchological features as a tool for specimen identification. The relatively high conchological variability found for *T.cilbanus*, together with the discovery of populations with intermediate conchological features between *T.cilbanus* and other closely related taxa, suggest that the determination of this species should be based on genetic criteria. Our molecular analyses demonstrate that *T.cilbanus* belongs to the *Iberus* genus, and thus, we proceed to update its taxonomic status to *Iberuscilbanus***comb. nov.**, and, thus, to consider *Tartessiberus* from now on as a junior synonym of *Iberus*.

## ﻿Introduction

The Iberian Peninsula is unquestionably a flora and fauna biodiversity hotspot (Orme et al. 2005) and contains an impressive diversity of land snails (Cadevall and Orozco 2016). The traditional determination of land snail species has typically been carried out based on morphological characters such as shell and genitalia. However, plenty of morphological traits are known to be of limited use in assessing land snail diversity at the species level (Gould and Woodruff 1986). It is not surprising that the use of genetic molecular tools has allowed for a better delimitation and more accurate understanding of biodiversity, in snail species too (Pfenninger et al. 2006). Molecular analyses are most useful in the detection of cryptic species that have passed unnoticed (Pfenninger and Magnin 2001; Nantarat et al. 2019; Liétor et al. 2024), and avoid cutting rather than lumping of species when purportedly different taxa are in fact only lineages without sufficient genetic differentiation to be considered separate species (Elejalde et al. 2008). Nevertheless, there are groups of taxa that have high genetic variability, and therefore caution is needed when interpreting genetic divergence.

Despite the importance of carrying out genetic analysis for species delimitation, still several land snail species, or even genera, are described solely based on anatomical and/or morphological approaches. A recent example is the description of *Tartessiberuscilbanus* Altaba & Ríos Jiménez (2021), a new monospecific genus endemic from southern Spain. The description and delimitation of this genus were entirely based on morphological and anatomical traits (genitalia, shell, and radula morphology), in comparison to closely related species of the tribe Allognathini. The fact that the morphology of this new species was intermediate between those of the genera *Iberus* Montfort, 1810 and *Allognathus* Pilsbry, 1888 directed the authors to create a new genus for the species. Despite morphological characters being useful for discerning within-population variance, they should be complemented with molecular analyses to complete taxonomic evidence when possible.

*Tartessiberuscilbanus* is linked to a number of snail populations located in the Sierra de Grazalema Natural Park (Cadiz Province, southwestern Spain), which were traditionally assigned to *Iberusloxanus* (A. Schmidt, 1855) because their shells fit within the pattern of variation of this species. However, *I.loxanus* exhibits a great conchological variation (Liétor 2014). Moreover, genetic analyses situated *I.loxanus* snails in phylogenetically separated clades, mixed with other supposed species (Elejalde et al. 2008). Elejalde et al.’s (2008) phylogenetic study not only showed that *I.loxanus* was a polyphyletic taxon, but also that several supposed species of *Iberus* (including *I.loxanus*) are different morphotypes of the same species [*I.marmoratus* (A. Férussac, 1821)]. However, Elejalde et al. (2008) did not include specimens attributable to *T.cilbanus* in their study. Hence, the phylogenetic position of this monospecific genus remained unknown.

The objective of this work is to analyse the phylogenetic position of *T.cilbanus*, providing molecular analyses of specimens sampled in various locations of its potential distribution area. The determination of its validity has important implications for cataloguing the Iberian land snail diversity and understanding the speciation processes in gastropods in the Iberian Peninsula.

## ﻿Materials and methods

### ﻿Field sampling

**Figure 1.**
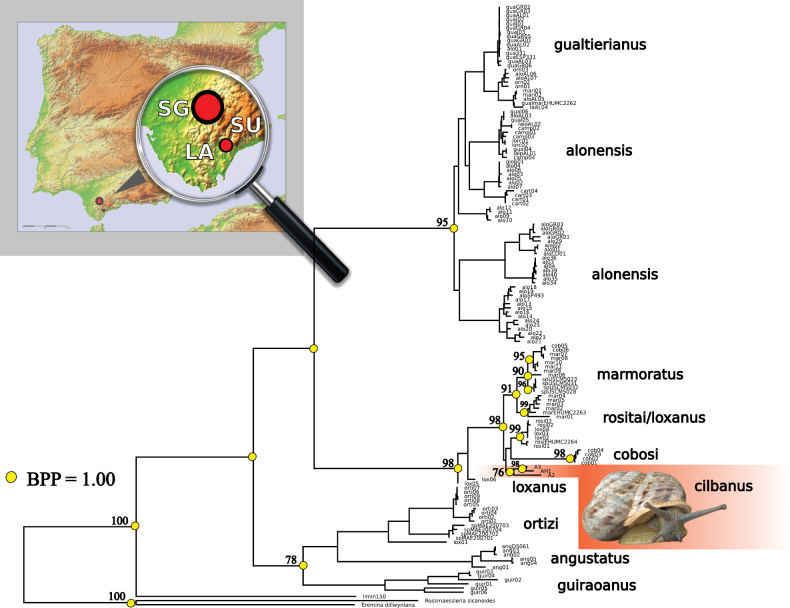
Top left: Map of the western provinces of Andalusia (Southern Spain) showing the geographic location (in red-filled circles) of the two known populations for *T.cilbanus*. Acronyms on map: SG (Sierra de Grazalema Natural Park, Cadiz), LA (Los Alcornocales Natural Park, Cadiz), SU (Sierra de la Utrera, Malaga). Right: maximum likelihood tree of *Iberus*. Values by nodes represent bootstrap values for the ML analyses (> 75%) and BI posterior probabilities (BPP = 1) (represented by yellow-filled circles) are shown for all major clades and for *T.cilbanus* and closely related taxa. *T.cilbanus* clade is shown in red.

We carried out a field sampling systematically covering all the calcareous mountain ranges of the potential distribution area of *T.cilbanus*, according to Altaba and Ríos Jiménez (2021). As a result, 11 field locations were sampled (Suppl. material 1: table S1) which allowed us to define a precise distribution area for the taxon (Fig. 1).

### ﻿Morphometrics

We measured 259 *T.cilbanus* shells. Measurements of shell morphometrics were conducted following López-Alcántara et al. (1985). Always the same researcher (JL) measured with a digital calliper (accuracy 0.01 mm): the largest and the smallest diameter (Ø) of the shell, shell height, and major and minor external Ø of the peristome. According to these data, we estimated the shell and peristome area, by considering that both the shell and the peristome may resemble an ellipse, applying the formula area = π × [(major Ø)/2] × [(minor Ø)/2]. On the basis of these measurements, we estimated a subsequent set of morphological ratios: shell height/major Ø of the shell (as an indicator of shell globosity, more globose shells having a higher ratio); major Ø of the shell/minor Ø of the shell (as an indicator of shell circularity, so that the closer this ratio is to unity, the greater the degree of circularity of the shell); major external Ø of the peristome/minor external Ø of the peristome (as an indicator of peristome circularity); percentage of the total surface of the shell occupied by the peristome [calculated as (peristome area x 100)/shell area].

We carried out statistical comparisons between morphometric measurements with those of the two taxa closely related both phylogenetically and geographically (*I.marmoratusloxanus* and *I.marmoratusmarmoratus*) with ANOVA tests when the variables were homoscedastic and normally distributed, otherwise using the Kruskal-Wallis test. In addition, a Principal Components Analysis (PCA) was carried out to determine the overlap in the morphospace between the populations of the described species and those of both *I.marmoratus* ssp. The variables used to place each population into the morphospace were the averages of the largest Ø and the height of the shells along with the average percentage of the total surface of the shells occupied by the peristome. These variables were shown to be adequate because more than 92% of the variance of the grouped data was explained by accumulating the first two principal components (PC).

### ﻿Molecular analysis

Three specimens (codes A2, A3, and AH1) were sacrificed by drowning and a tissue sample was extracted for molecular analyses, stored in absolute ethanol and maintained at -20 °C. Specimen A3 was collected 660 m north from the type locality shown by Altaba and Ríos Jiménez (2021).

Genomic DNA was extracted using QIAGEN DNeasy Blood and Tissue Kit (Qiagen, Hilden, Germany) according to the manufacturer’s protocol. The total alignment comprises all known *Iberus* sequences from Genbank (*N* = 141) including *Iberellus* sp. and two outgroup taxa, (*Rossmaessleriasicanoides* (Kobelt, 1881) and *Ereminadillwyniana* (L. Pfeiffer, 1853) (Suppl. material 1: table S2).

We firstly used the primers LCO and HCO (Folmer et al. 1994) to amplify the mitochondrial cytochrome oxidase I (COI) gene, but amplifications were sometimes problematic, and therefore we designed specific primers for *Iberus* (F: ATAAYGTTATTGTTACTGCYCATGCATTYG, R: AGATGTTGRTAYARAATRGGRTCYCC ~600 pb). We used primers (F: CGCCTGTTTATCAAAAACAT, R: CCGGTCTGAACTCAGATCACGT) from Palumbi (1996) to amplify a 480 bp of the mitochondrial 16S ribosomal RNA (16S rRNA), and primers (F: CTAGCTGCGAGAATTAATGTGA, R: ACTTTCCCTCACGGTACTTG) from Wade et al. (2006) to amplify and sequence a ~900 pb fraction of the nuclear gene large ribosomal subunit (LSU). Sequences were edited with Sequencher v.5.4.6 (Gene Codes Corporation, Ann Arbor, MI, USA), and checked for potential contaminants using GenBank’s BLASTn search (Altschul et al. 1990). Sequences were edited in Seaview v.4.2.11 (Gouy et al. 2010) and aligned with MAFFT (Katoh et al. 2002) in the CIPRES platform (Miller et al. 2010).

Phylogenetic tree reconstructions for the three concatenated gene fragments (total length 1984 bp) were performed using maximum likelihood (ML) and Bayesian inference (BI), through RAxML v.7.0.4 (Silvestro and Michalak 2012) and MrBayes v.3.2, (Ronquist and Huelsenbeck 2003), respectively. The Akaike Information Criterion (AICc) and partition scheme was implemented in PartitionFinder v.2.1.1 (Lanfear et al. 2016), using a ´greedy´search (Lanfear et al. 2012) to select the best fit evolutionary model for each partition. The re­sulting models and partitions were GTR+I+G (COI pos1), F81+I (COI pos2), GTR+I+G (COI pos3), GTR+I+G (16S rRNA) and HKY+G (LSU).

From the BI analysis, two independent runs (each with four Markov chains for 10 × 10^7^ generations) were performed. Trees and parameters were sampled every 1000 generations. A majority-rule consensus tree was estimated by combining results from duplicated analyses, after discarding 25% of the total samples as burn-in. ML searches were conducted under GTRGAMMA and support was assessed by using 1000 bootstrapped replicates. All phylogenetic analyses were performed in the CIPRES platform (Miller et al. 2010). The consensus tree was visualised and rooted using FigTree v.1.4.4 (Rambaut 2018), and later prepared as a graphic with the software Inkscape v.1.0.1 (http://www.inkscape.org).

## ﻿Results

### ﻿Phylogenetic analyses and genetic distances

The phylogenetic analyses recovered three well-supported clades for the genus *Iberus* with *Tartessiberus* included within the tree topology, a clear indication that this later genus cannot be valid. Sequences of *T.cilbanus* specimens were grouped in the centre clade, with *I.rositai*, *I.loxanus*, *I.marmoratus* and *Iberus* sp. (Fig. 1). The *T.cilbanus* clade was strongly supported in both the ML and BI analyses. Analyses of the nuclear gene tree placed the three samples within the same *Iberus* clade (data not shown) as the mitochondrial data did. GenBank blast searches of the nuclear fragment matched 99.81% with *I.rositai*, *I.marmoratus*, *I.loxanus* and *I.cobosi*.

Genetic divergence between *T.cilbanus* and the rest of the closely associated taxa remained high, with a minimum divergence of 7.5% and a maximum of 10.9% for the COI and 3% and 5.8% for the 16S rRNA gene fraction (Table 1). Genetic divergence within individuals from the *T.cilbanus* clade was high, as the A2 and A3+AH1 had a genetic distance between them of 7.1% and 3.4% for the COI and 16S rRNA, respectively. Overall, the mean genetic divergence within *T.cilbanus* was 5.6% (COI) and 2.3% (16S rRNA). Meanwhile, within other closely related species, genetic divergences were: *I.cobosi*, 0.9% (COI), 0.13% (16S rRNA); *I.loxanus* 05+06, 0.9% (COI), 1.3% (16S rRNA); *I.marmoratus*+sp, 4.1% (COI), 2.4% (16S rRNA); *I.rositai*+*loxanus*, 1.8% (COI), 0.09% (16S rRNA).

**Table 1.** *P*-uncorrected distances for the taxa of the clade closely associated with *T.cilbanus*, COI (lower matrix) and 16S rRNA (upper matrix).

	*T.cilbanus*	*I.cobosi*	*I.loxanus*	*I.marmoratus*	*I.rositai*/*loxanus*
***T.cilbanus***	–	5.80%	3.07%	4.99%	3.25%
***I.cobosi***	10.90%	–	4.74%	5.76%	5.04%
***I.loxanus***	10.45%	12.73%	–	4.28%	2.58%
***I.marmoratus***	8.23%	11.13%	10.65%	–	4.19%
***I.rositai*/*loxanus***	7.48%	10.09%	10.18%	7.73%	–

### ﻿Distribution

As expected, most locations for *T.cilbanus* were from the Cadiz Province. Nevertheless, a new locality was found in the Sierra de la Utrera massif (province of Malaga, southern Spain), a karstic habitat ecologically analogous to that of its main distribution region in the Grazalema Natural Park (Fig. 1). The specimens from Sierra de la Utrera showed shell sizes below standard for the species (318 mm^2^ of average shell area (*N* = 19), significantly lower than 424 mm^2^ for the remaining *T.cilbanus* (*N* = 240); p-value = 0.000009 for one-way ANOVA plus post hoc Tukey test). Moreover, our field samplings improved the knowledge of the distribution of this species with new locations that extend its distribution range to approximately 200 km^2^. The altitudinal range is also more precisely determined, to the interval from 314 to 1257 m a.s.l. (Suppl. material 1: table S1).

### ﻿Morphology

**Figure 2.**
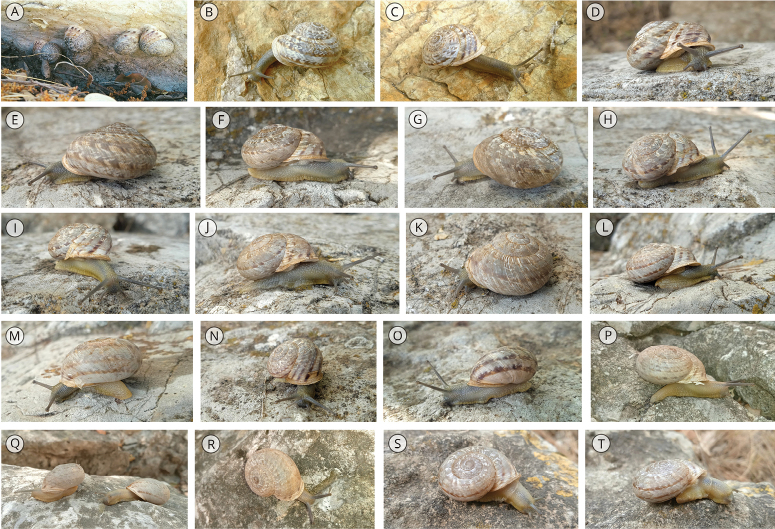
Live specimens of *T.cilbanus* from Cadiz Province photographed in situ **A–I** Grazalema town ring road, Grazalema Natural Park **J–O** Benaocaz, Grazalema Natural Park **P–T** next to the Caldereto neighborhood, Ubrique, Grazalema Natural Park.

**Figure 3.**
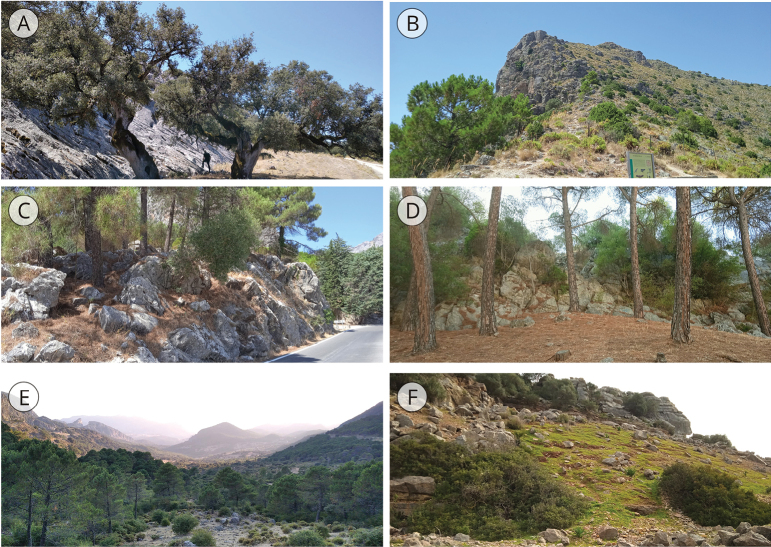
Habitats of *T.cilbanus*. **A–E** Grazalema Natural Park, Cadiz Province (**A** Llanos del Apeo, Los Alamos **B** Puerto de las Palomas **C** Grazalema town ring road **D** Caldereto neighborhood, Ubrique **E** ‘El Cintillo’ viewpoint, Benaocaz) **F** Sierra de la Utrera, Manilva, Casares, Malaga Province.

Suppl. material 1: fig. S1 shows a series of specimens of *T.cilbanus* covering its range of conchological variability, which is complemented with images of living specimens in situ (Fig. 2) and their habitats (Fig. 3).

**Figure 4.**
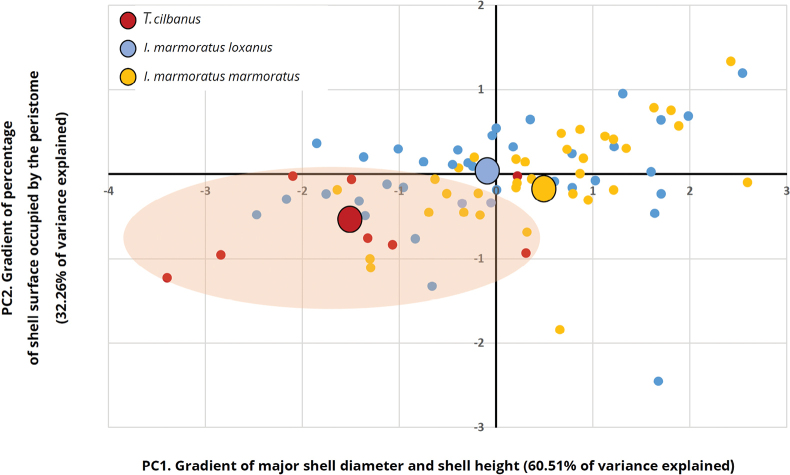
Distribution of *T.cilbanus* (8 localities), *I.marmoratusloxanus* (35 localities) and *I.marmoratusmarmoratus* (36 localities) in the bidimensional space generated by the two first PC of a PCA analysis. Each point in the graph represents a single sampling locality. Coordinates of centroids for each species have been calculated as the average X and Y coordinates of the points included in the corresponding clouds. *T.cilbanus* cloud has been highlighted in light red.

The first factor of the PCA (PC1, Fig. 4) combined major shell Ø and shell height, thus being assignable to a gradient of shell size which increases from left to right along the x-axis. PC1 best captured the morphological variability of the shells, with 60.51% of the variance of the morphometric data. The second factor (PC2, Fig. 4), a gradient of the percentage of the shell surface that is occupied by the peristome (increasing from bottom to top along the y-axis), grouped the populations more weakly, explaining 32.26% of the data variance. The PCA showed that *T.cilbanus* occupies a position in the two-dimensional space separated from the subspecies of the *I.marmoratus* complex, which show very similar shells. Still, some overlap between *T.cilbanus* and the other two taxa may be found (Fig. 4).

Suppl. material 1: table S3 summarises the morphometric data of 259 shells of *T.cilbanus* from eight sampling locations. Most morphometric parameters measured in the shells of *T.cilbanus* significantly exceeded those of the two subspecies of the *I.marmoratus* complex, which are phylogenetically and geographically closely related. The shells of *T.cilbanus* were wider, taller, more globose, and with a larger area than those of the *I.marmoratus* ssp. The peristome of *T.cilbanus* was larger and, therefore, had a greater area, which is also manifested in a greater relative area with respect to the total area of the shell, in comparison to *I.marmoratus* ssp. The only morphometric parameters that did not show statistical differences among the three taxa compared were the circularity of both the shells and the peristomes (Suppl. material 1: table S4).

**Figure 5.**
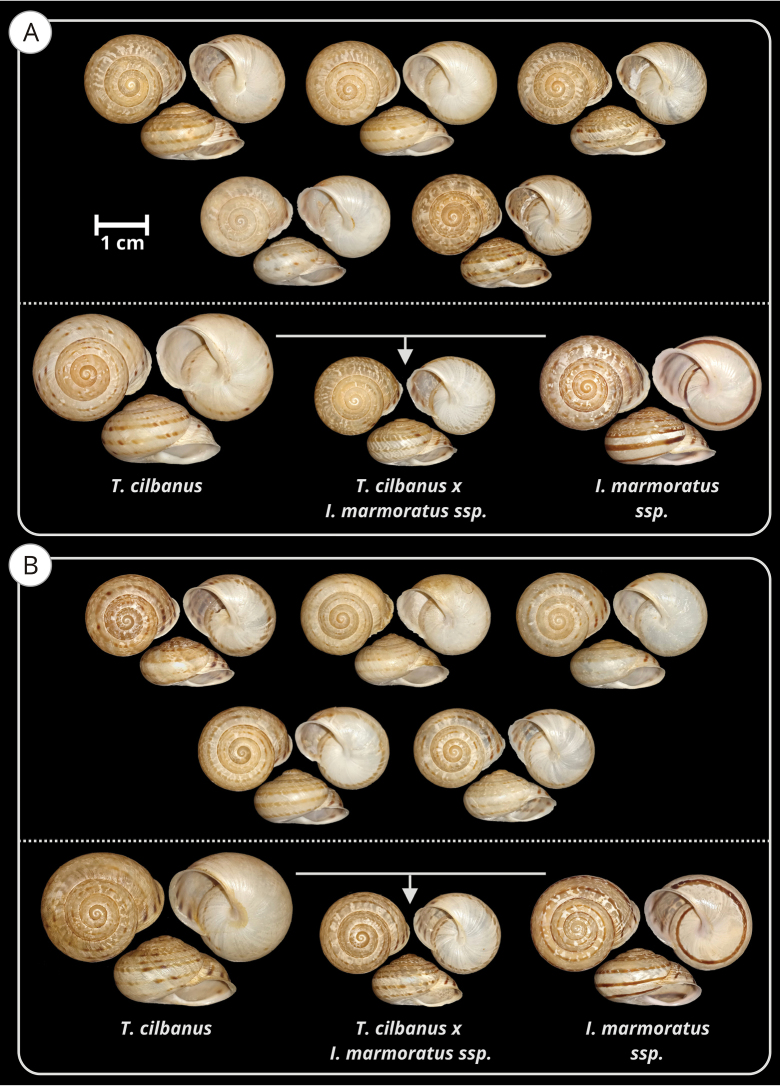
Photographic series of intermediate specimens between *T.cilbanus* and *I.marmoratus* ssp. **A** Hermitage of Virgencita, Algodonales, Sierra de Lijar (Cadiz Province) **B** Sierra Crestellina, Casares (Malaga Province). Below each photographic series, a tentative composition with the parents and an intermediate specimen in a central position is displayed. Selected shells of *I.marmoratus* ssp. come from the closest locations where sampling material was available: Cueva del Gato, Benaojan (Malaga Province) for series A and Gaucin Castle (Malaga Province) for series B.

During the sampling, we found populations composed of dwarf-sized specimens with intermediate conchological characteristics between *T.cilbanus* and other taxa of the *I.marmoratus* complex that surround the Grazalema Natural Park. These populations were found in the distribution margins of *T.cilbanus*, pointing to possible genetic introgression in the north (Algodonales, Cadiz Province), as well as in the south (Casares, Malaga Province). Fig. 5 shows some shells of specimens from both populations. The major and minor average shell and peristome diameters, as well as the average shell height, were found to be significantly lower in the two aforementioned dwarf populations than in *T.cilbanus* (p-value < 0.00001, Kruskal Wallis plus 2-tailed multiple comparison H test).

## ﻿Discussion

Altaba and Ríos Jiménez (2021) defined the genus *Tartessiberus* on the basis of morphological and anatomical traits (genitalia, shell and radula). Our genetic study on *Tartessiberus* is yet another example of how genetic tools may further contribute to define taxonomic levels in snails (e.g., Gould and Woodruff 1986; Pfenninger and Magnin 2001; Haase and Bisenberger 2003; Teshima et al. 2003; Pfenninger et al. 2006; Nantarat et al. 2019). In our study, the three sequenced individuals ascribed to *T.cilbanus* grouped within the genus *Iberus*. Therefore, we can unequivocally affirm that snails believed to be *T.cilbanus* are indeed *Iberus* land snails. Furthermore, the genetic distances with other lineages within the closely related clades and its monophyly, with no shared haplotypes to other taxa, suggest the validity of *Iberuscilbanus* comb. nov. (*I.cilbanus* hereafter). However, the notable intraspecific divergence found for *I.cilbanus* suggests the need for subsequent studies on a larger number of samples to determine whether we are dealing with one or several taxa.

The position of *I.cilbanus* as an independent lineage rules out that this clade could be mistaken for any of its closely related species. Our findings, consequently, provide a study case highlighting the importance of genetic analysis to correctly assign taxonomic value when describing species or even genera, although Altaba and Ríos Jiménez (2021) did correctly describe a new species without molecular tools.

In addition to the phylogenetic position, we rely on genetic divergence to ascertain the high genetic differentiation between *I.cilbanus* and its sister clade (Fig. 1). The genetic threshold for considering separated species may be, to some degree, arbitrary. Davison et al. (2009) proposed a 4% threshold for establishing limits between land snail species (with a relatively high rate of error). However, Köhler and Johnson (2012) suggested at least 6% genetic distance for the COI based on their study in insular land snails, which showed up to 6% variance within species and at least 6% variance between species inhabiting different islands. Moreover, for molluscs, the divergence between congeneric species typically is over 8% (67.5% of cases), with only 15% of pairs of congeneric species showing distances between 4 and 8% (Hebert et al. 2003). But there are known exceptions in some groups and, therefore, this data alone should be treated with caution. Despite these numbers, we are aware that there is no cut-off point to species delimitation based on genetic distances per se, and we enter the conundrum of ‘how long is a piece of string’. Nevertheless, the presence of a clear, strongly supported clade, morphologically differentiated from other *Iberus* species and subspecies, the high genetic divergence, as well as moderate geographical separation, firmly support the validity of a distinct *Iberus* species (i.e., *I.cilbanus*). *Iberuscilbanus* showed a morphology on average well differentiated from *I.marmoratus* spp. (see Fig. 5), the nearest taxon geographically speaking. Their distribution is also separated, although there are a few contact areas. Its reduced distribution range (200 km^2^) and the existence of some fragmented isolated populations (in Sierra de la Utrera) suggest that some conservation considerations might be necessary for this species.

The existence of the genus *Tartessiberus* would not only imply an unusually young genus (~ 5 Ma versus *Iberus* at 18. 5 Ma; Neiber et al. 2021) but also the paraphyly of *Iberus*, suggesting the need for immense taxonomic changes. One other genus, *Pseudotachea* C. R. Boettger, 1909, remains positioned within the *Iberus* clade though Neiber et al. (2021) suggest its synonymization with *Iberus*. Therefore, with *Tartessiberus* and *Pseudotachea* synonymized with *Iberus*, the latter remains monophyletic, which implies an ancient evolutionary lineage and origin for the Iberian Peninsula.

Our field observations and captive breeding trials (unpublished data) have found that individuals and populations of different species of the genus *Iberus* tend to show dwarfism tendencies as a possible consequence of hybridization. Further studies will be necessary to determine if the smaller population of Sierra de la Utrera is undergoing a process of introgression by *I.marmoratusmarmoratus* or, alternatively, if the small size is a local adaptive response or a symptom of phenotypic plasticity. Further genetic sequencing will corroborate possible hybridization between these species.

## ﻿Additional information

### Author ORCIDs

Michael J. Jowers https://orcid.org/0000-0001-8935-5913

José Liétor https://orcid.org/0009-0009-5877-6550

Antonio R. Tudela https://orcid.org/0000-0002-9402-6345

Pedro A. Jódar https://orcid.org/0009-0001-1691-745X

Inés Galán-Luque https://orcid.org/0000-0003-2356-9374

Gregorio Moreno-Rueda https://orcid.org/0000-0002-6726-7215

### Data availability

All data generated or analysed during this study are included in this published article (Supporting information).

## Supplementary materials

Supplementary material 1 Supporting information Data type docx Explanation note **figure S1.** Photographic series showing the range of variability for the shells of *T.cilbanus* (Cadiz): 1–11: Grazalema town ring road, Grazalema Natural Park; 12–20: Next to the Caldereto neighborhood, Ubrique, Grazalema Natural Park; 21–24: Llanos del Apeo, Los Alamos, Grazalema Natural Park; 25–39: `El Cintillo´ viewpoint, Benaocaz, Grazalema Natural Park; (Malaga); 40: Sierra de la Utrera, Manilva, Casares. **table S1.** Sampling locations for *T.cilbanus*. **table S2.** Samples used in the phylogenetic analyses. GenBank voucher abbreviations, species names, localities, coordinates and GenBank accessions. **table S3.** Morphometric parameters and ratios measured for *T.cilbanus* (*N* = 259). **table S4.** Morphometric comparisons between *T.cilbanus* and the two taxa of the *marmoratus* complex which inhabit the surrounding areas. K: Kruskal Wallis plus 2-tailed multiple comparison H test; A: one-way ANOVA plus post hoc Tukey test (HSD) for the comparisons between *T.cilbanus* and *I.marmoratusmarmoratus* and *I.marmoratusloxanus*, respectively; ns: non-significant. https://binary.pensoft.net/file/1049017This dataset is made available under the Open Database License (http://opendatacommons.org/licenses/odbl/1.0/). The Open Database License (ODbL) is a license agreement intended to allow users to freely share, modify, and use this Dataset while maintaining this same freedom for others, provided that the original source and author(s) are credited.Michael J. Jowers, José Liétor, Antonio R. Tudela, Pedro A. Jódar, Inés Galán-Luque, Gregorio Moreno-Rueda
