# Supplementary material for: ﻿Phylogenetic evidence suggests the non-validity of the Iberian land snail genus Tartessiberus and confirms its synonymy with Iberus (Helicidae)
Source: Zookeys. 2024 May 14;1201:219–31. doi: 10.3897/zookeys.1201.117318 (PMC11109508; doi:10.3897/zookeys.1201.117318)
Supplement: Supplementary material 1 — Supporting information [file zookeys-1201-219_article-117318__-s001.docx]

**Supplementary Material**

**Supplementary Figure S1**

Photographic series showing the range of variability for the shells of *T. cilbanus* (Cadiz): 1-11: Grazalema town ring road, Grazalema Natural Park; 12-20: Next to the Caldereto neighborhood, Ubrique, Grazalema Natural Park; 21-24: Llanos del Apeo, Los Alamos, Grazalema Natural Park; 25-39: `El Cintillo´ viewpoint, Benaocaz, Grazalema Natural Park; (Malaga); 40: Sierra de la Utrera, Manilva, Casares.


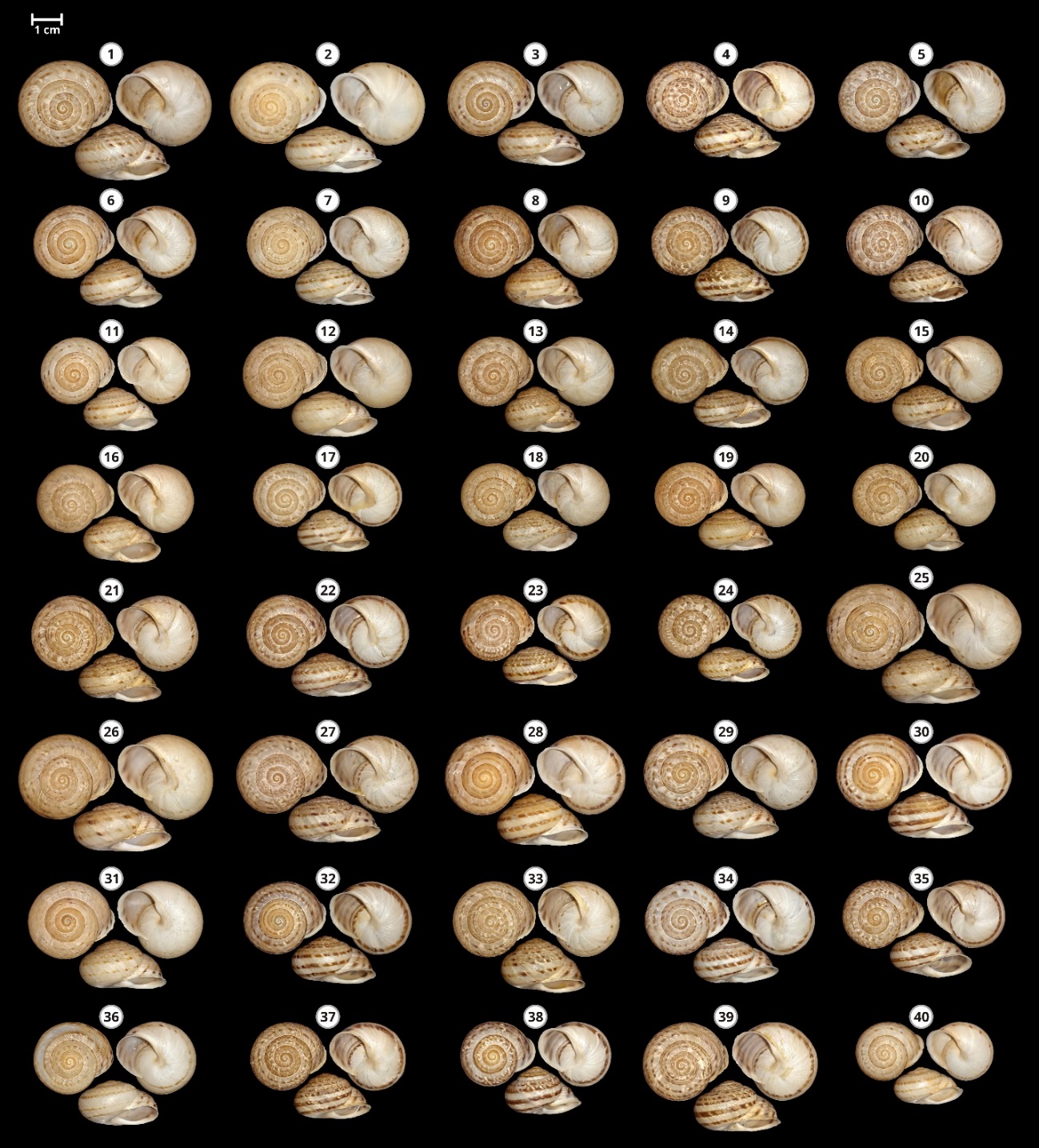


**Table S1**

Sampling locations for *T. cilbanus*.

| Province | Location | Latitude  (N) | Longitude  (W) | Altitude  (m a.s.l.) |
| --- | --- | --- | --- | --- |
| Cadiz (Grazalema Natural Park) | Grazalema ring road | 36° 45' 30'' | -5° 22' 19'' | 898 |
|  | Grazalema ring road | 36° 45' 25'' | -5° 21' 58'' | 861 |
|  | Llanos del Apeo, Los Alamos | 36° 43' 32'' | -5° 19' 41'' | 855 |
|  | `El Cintillo´ Viewpoint, Benaocaz | 36° 41' 04'' | -5° 25' 23'' | 760 |
|  | Puerto de las Palomas, Gaidovar, Zahara de la Sierra | 36° 47' 16'' | -5° 22' 35'' | 1192 |
|  | Caldereto neighborhood, Ubrique | 36° 40' 24'' | -5° 26' 40'' | 368 |
|  | Los Cañitos recreational area, El Bosque | 36° 44' 39'' | -5° 30' 12'' | 314 |
|  | Benamahoma | 36° 45' 38'' | -5° 27' 45'' | 513 |
|  | Near Zahara de la Sierra castle | 36° 50' 22'' | -5° 23' 28'' | 539 |
|  | Near Puerto del Boyar | 36° 45' 52'' | -5° 23' 19'' | 1257 |
| Malaga | Sierra de la Utrera, Manilva, Casares | 36° 23' 52'' | -5° 16' 25'' | 323 |

**Table S2**

Samples used in the phylogenetic analyses. GenBank voucher abbreviations, species names, localities, coordinates and GenBank accessions.

| **CODE / voucher** | **SPECIES** | **LOCALITY** | | **GenBank accession number** | | |
| --- | --- | --- | --- | --- | --- | --- |
|  |  | **Latitude** | **Longitude** | **COI** | **16S rRNA** | **LSU** |
| A2 | *Iberus cilbanus* | 36º 43' 31.5'' N | 5º 19' 41.1'' W | PP384386 | PP379195 | PP384506 |
| A3 | *Iberus cilbanus* | 36º 45' 29.3'' N | 5º 22' 19.3'' W | PP384387 | PP379196 | PP384507 |
| AH1 | *Iberus cilbanus* | 36° 40' 24.4" N | 5° 26' 40.2'' W | PP384388 | PP379197 | PP384508 |
| **Genbank CODE / Voucher** | **SPECIES** | **LOCALITY** | | **GenBank accession number** | | |
|  |  | **UTM** | | **COI** | **16S rRNA** | **LSU** |
| I.angustatus05 | *Iberus angustatus* | 30SVG37 | | EF440242 | EF440192 | - |
| I.angustatus04 | *Iberus angustatus* | 30SVG17 | | EF440241 | EF440191 | - |
| I.angustatus03 | *Iberus angustatus* | 30SVG52 | | EF440240 | EF440190 | MW817736 |
| I.angus_MVHN-091012DS06-1 | *Iberus angustatus* | - | | KC693589 | - | - |
| I.angustatus02 | *Iberus angustatus* | 30SVG57 | | EF440239 | EF440189 | - |
| I.angustatus01 | *Iberus angustatus* | 30SVG67 | | EF440238 | EF440188 | - |
| Iberus sp. MAE-2007-03 | *Iberus giennensis* | 30SVG23 | | EF440274 | EF440224 | - |
| Iberus sp. MAE-2007-02 | *Iberus giennensis* | 30SVG14 | | EF440273 | EF440223 | - |
| Iberus sp. MAE-2007-01 | *Iberus giennensis* | 30SVG14 | | EF440272 | EF440222 | MW817751 |
| Iberus sp. MAE-2007-04 | *Iberus giennensis* | 30SVG22 | | EF440275 | EF440225 | - |
| I.ortizi09 | *Iberus ortizi* | 30SUG74 | | EF440284 | EF440234 | - |
| I.ortizi08 | *Iberus ortizi* | 30SUG74 | | EF440283 | EF440233 | MW817749 |
| I.ortizi07 | *Iberus ortizi* | 30SUG84 | | EF440282 | EF440232 | - |
| I.ortizi06 | *Iberus ortizi* | 30SUG74 | | EF440281 | EF440231 | - |
| I.ortizi05 | *Iberus ortizi* | 30SUG74 | | EF440280 | EF440230 | - |
| I.ortizi04 | *Iberus ortizi* | 30SUG84 | | EF440279 | EF440229 |  |
| I.ortizi03 | *Iberus ortizi* | 30SUG84 | | EF440278 | EF440228 |  |
| I.ortizi02 | *Iberus ortizi* | 30SUG84 | | EF440277 | EF440227 | - |
| I.ortizi01 | *Iberus ortizi* | 30SUG84 | | EF440276 | EF440226 | - |
| I.loxanus01 | *Iberus antikarianus* | 30SUF79 | | EF440255 | EF440205 | MW817741 |
| I.guiraoanus06 | *Iberus guiraoanus* | 30SWG18 | | EF440254 | EF440204 | - |
| I.guiraoanus05 | *Iberus guiraoanus* | 30SWG192 | | EF440253 | EF440203 | - |
| I.guiraoanus04 | *Iberus guiraoanus* | 30SWH21 | | EF440252 | EF440202 | - |
| I.guiraoanus03 | *Iberus guiraoanus* | 30SWG09 | | EF440251 | EF440201 | - |
| I.guiraoanus02 | *Iberus guiraoanus* | 30SWG09 | | EF440250 | EF440200 | - |
| I.guiraoanus01 | *Iberus guiraoanus* | 30SWH33 | | EF440249 | EF440199 | MW817746 |
| Iberus sp. | *Iberus sp* | PN Alcornocales | | MF982904 | - | - |
| Iberus sp. | *Iberus sp* | PN Alcornocales | | MF982905 | - | - |
| Iberus sp. | *Iberus sp* | PN Alcornocales | | MF982902 | - | - |
| Iberus sp. | *Iberus sp* | PN Alcornocales | | MF982903 | - | - |
| I. cobosi06 | *Iberus cobosi* | 30SUF48 | | EF440248 | EF440198 | - |
| I. cobosi05 | *Iberus cobosi* | 30SUF48 | | EF440247 | EF440197 | - |
| I.marmoratus08 | *Iberus marmoratus* | 30SUF58 | | EF440268 | EF440218 | - |
| I.marmoratus07 | *Iberus marmoratus* | 30SUF58 | | EF440267 | EF440217 | - |
| I.marmoratus11 | *Iberus marmoratus* | 30SUF48 | | EF440271 | EF440221 | - |
| I.marmoratus10 | *Iberus marmoratus* | 30SUF49 | | EF440270 | EF440220 | - |
| I.marmoratus09 | *Iberus marmoratus* | 30SUF76 | | EF440269 | EF440219 | - |
| I.marmoratus06 | *Iberus marmoratus* | 30SUG09 | | EF440266 | EF440216 | - |
| I.marmoratus05 | *Iberus marmoratus* | 30STF96 | | EF440265 | EF440215 | - |
| I.marmoratus04 | *Iberus marmoratus* | 30SUF15 | | EF440264 | EF440214 |  |
| I.marmoratus03 | *Iberus marmoratus* | 30SUF34 | | EF440263 | EF440213 | - |
| I.marmoratus02 | *Iberus marmoratus* | 30SUF24 | | EF440262 | EF440212 |  |
| I.marmEoratusHUMC2263 | *Iberus marmoratus* | 36°26'56'' N -005°12'33'' E | | MW803160 | MW817709 | MW817748 |
| marmoratus-01 | *Iberus marmoratus* | 30SUF17 | | EF440261 | EF440211 | - |
| I.rositai02 | *Iberus rositai* | 30SUF69 | | EF440286 | EF440236 | - |
| I.rositai03 | *Iberus rositai* | 30SUF69 | | EF440287 | EF440237 | - |
| I.loxanus04 | *Iberus loxanus* | 30SUF69 | | EF440258 | EF440208 | - |
| I.loxanus03 | *Iberus loxanus* | 30SUF69 | | EF440257 | EF440207 | - |
| I.loxanus02 | *Iberus loxanus* | 30SUF69 | | EF440256 | EF440206 | - |
| I.rositai01 | *Iberus rositai* | 30SUF69 | | EF440285 | EF440235 | - |
| IrositaiEHUMC2264 | *Iberus rositai* | 36º56'49'' N -004º33'48'' E | | MW803161 | MW817711 | MW817750 |
| I.cobosi04 | *Iberus cobosi* | 30SUF48 | | EF440246 | EF440196 | - |
| I.cobosi03 | *Iberus cobosi* | 30SUF48 | | EF440245 | EF440195 | - |
| I.cobosi02 | *Iberus cobosi* | 30SUF48 | | EF440244 | EF440194 | - |
| I.cobosi01 | *Iberus cobosi* | 30SUF48 | | EF440243 | EF440193 | MW817742 |
| I.loxanus06 | *Iberus loxanus* | 30SUG91 | | EF440260 | EF440210 | - |
| I.loxanus-05 | *Iberus loxanus* | 30SVF57 | | EF440259 | EF440209 | MW817747 |
| Ialo25 | *Iberus gualterianus alonensis* | 30SUG84 | | AY928559 | AY928587 | - |
| Ialo24 | *Iberus gualterianus alonensis* | 30SXH71 | | DQ822148 | DQ822098 | - |
| Ialo23 | *Iberus gualterianus alonensis* | 30SXH62 | | DQ822147 | DQ822097 | - |
| Ialo22 | *Iberus gualterianus alonensis* | 30SYH26 | | DQ822146 | DQ822096 | - |
| Ialo21 | *Iberus gualterianus alonensis* | 30SYH05 | | DQ822145 | DQ822095 | - |
| Ialo20 | *Iberus gualterianus alonensis* | 30TWM58 | | DQ822144 | DQ822094 | - |
| Ialo19 | *Iberus gualterianus alonensis* | 30SXH51 | | DQ822143 | DQ822093 | - |
| Ialo18 | *Iberus gualterianus alonensis* | 31TCF23 | | DQ822142 | DQ822092 | - |
| Ialonensis_SP493 | *Iberus alonensis* | 41°06'30'' N 000°52'33'' E | | MW803158 | MW817696 | MW817735 |
| Ialo17 | *Iberus gualterianus alonensis* | 30TYL22 | | DQ822141 | DQ822091 | - |
| Ialo15 | *Iberus gualterianus alonensis* | 30TWL77 | | DQ822139 | DQ822089 | - |
| Ialo13 | *Iberus gualterianus alonensis* | 31TBF60 | | DQ822137 | DQ822087 | - |
| Ialo16 | *Iberus gualterianus alonensis* | 30TYL48 | | DQ822140 | DQ822090 | - |
| Ialo14 | *Iberus gualterianus alonensis* | 30SWH48 | | DQ822138 | DQ822088 | - |
| Ialo12 | *Iberus gualterianus alonensis* | 30TUJ09 | | DQ822136 | DQ822086 | MW817740 |
| Ialo11 | *Iberus gualterianus alonensis* | 30SWF59 | | DQ822135 | DQ822085 | - |
| Ialo10 | *Iberus gualterianus alonensis* | 30SWF58 | | DQ822134 | DQ822084 | - |
| Ialo09 | *Iberus gualterianus alonensis* | 30SWF87 | | DQ822133 | DQ822083 | - |
| Iglob01 | *Iberus gualterianus globosus* | 30SWG86 | | DQ822163 | DQ822113 | - |
| Ialo07 | *Iberus gualterianus alonensis* | 30SXG17 | | DQ822131 | DQ822081 | MW817743 |
| Ialo06 | *Iberus gualterianus alonensis* | 30SWG15 | | DQ822130 | DQ822080 | - |
| Ialo05 | *Iberus gualterianus alonensis* | 30SWG48 | | DQ822129 | DQ822079 | - |
| Ialo04 | *Iberus gualterianus alonensis* | 30SWG15 | | DQ822128 | DQ822078 | - |
| Ialo03 | *Iberus gualterianus alonensis* | 30SXG17 | | DQ822127 | DQ822077 | - |
| Ialo02 | *Iberus gualterianus alonensis* | 30SWG26 | | DQ822126 | DQ822076 | - |
| Icart04 | *Iberus gualterianus carthaginiensis* | 30SXG76 | | DQ822162 | DQ822112 | - |
| Icart03 | *Iberus gualterianus carthaginiensis* | 30SXG76 | | DQ822161 | DQ822111 | - |
| Icart02 | *Iberus gualterianus carthaginiensis* | 30SXG76 | | DQ822160 | DQ822110 | - |
| Icart01 | *Iberus gualterianus carthaginiensis* | 30SXG76 | | DQ822159 | DQ822109 | MW817738 |
| IaAL03 | *Iberus gualterianus* | 30SWG85 | | [AY928554](https://www.ncbi.nlm.nih.gov/nuccore/AY928554) | [AY928582](https://www.ncbi.nlm.nih.gov/nuccore/AY928582) | - |
| Igual06 | *Iberus gualterianus gualterianus* | 30SWG93 | | DQ822166 | DQ822116 | - |
| Igual05 | *Iberus gualterianus gualterianus* | 30SWG93 | | DQ822165 | DQ822115 | - |
| Icamp03 | *Iberus gualterianus campesinus* | 30SXG13 | | DQ822157 | DQ822107 | - |
| Icamp01 | *Iberus gualterianus campesinus* | 30SWG62 | | DQ822155 | DQ822105 | MW817737 |
| Ilorc02 | *Iberus gualterianus lorcanus* | 30SXG25 | | DQ822168 | DQ822118 | - |
| Ilorc01 | *Iberus gualterianus lorcanus* | 30SXG25 | | DQ822167 | DQ822117 | - |
| IaAL02 | *Iberus gualterianus* | 30SXG02 | | [AY928553](https://www.ncbi.nlm.nih.gov/nuccore/AY928553) | [AY928581](https://www.ncbi.nlm.nih.gov/nuccore/AY928581) | - |
| Icamp02 | *Iberus gualterianus campesinus* | 30SXG02 | | DQ822156 | DQ822106 | - |
| IaAL01 | *Iberus gualterianus* | 30SWG82 | | AY928552 | AY928580 | - |
| Igual04 | *Iberus gualterianus gualterianus* | 30SWG93 | | DQ822164 | DQ822114 | - |
| Icamp04 | *Iberus gualterianus campesinus* | 30SWG92 | | DQ822158 | DQ822108 | - |
| IaJ04 | *Iberus gualterianus* | 30SVG98 | | AY928567 | AY928595 | - |
| IaJ03 | *Iberus gualterianus* | 30SVG68 | | AY928566 | AY928594 | - |
| Ialo36 | *Iberus gualterianus alonensis* | 30SWF07 | | DQ822152 | DQ822102 | - |
| Ialo40 | *Iberus gualterianus alonensis* | 30SWF07 | | DQ822154 | DQ822104 | - |
| Ialo39 | *Iberus gualterianus alonensis* | 30SWF07 | | DQ822153 | DQ822103 | - |
| Ialo35 | *Iberus gualterianus alonensis* | 30SVG72 | | DQ822152 | DQ822102 | - |
| Ialo34 | *Iberus gualterianus alonensis* | 30SWF07 | | DQ822151 | DQ822101 | - |
| IaJ02 | *Iberus gualterianus* | 30SVG37 | | AY928565 | AY928593 | - |
| IaJ01 | *Iberus gualterianus* | 30SVG37 | | AY928564 | AY928592 | - |
| IaCO01 | *Iberus gualterianus* | 30SUG85 | | AY928559 | AY928587 | - |
| IgGR04 | *Iberus gualterianus* | 30SVG32 | | AY928574 | AY928602 | - |
| IgGR03 | *Iberus gualterianus* | 30SVG32 | | AY928573 | AY928601 | - |
| IgGR02 | *Iberus gualterianus gualterianus* | 30SVG32 | | AY928572 | AY928600 | MW817739 |
| IaGR01 | *Iberus gualterianus* | 30SVF57 | | AY928560 | AY928588 | - |
| Ialo29 | *Iberus gualterianus alonensis* | 30SVF57 | | AY928560 | AY928588 | - |
| IaAL07 | *Iberus gualterianus* | 30SWF27 | | [AY928558](https://www.ncbi.nlm.nih.gov/nuccore/AY928558) | [AY928586](https://www.ncbi.nlm.nih.gov/nuccore/AY928586) | - |
| IaAL06 | *Iberus gualterianus* | 30SWF37 | | [AY928557](https://www.ncbi.nlm.nih.gov/nuccore/AY928557) | [AY928585](https://www.ncbi.nlm.nih.gov/nuccore/AY928585) | - |
| Iorn03 | *Iberus gualterianus ornatissimus* | 30SWF39 | | DQ822173 | DQ822123 | - |
| Iorn02 | *Iberus gualterianus ornatissimus* | 30SWF39 | | DQ822172 | DQ822122 | - |
| Iorn01 | *Iberus gualterianus ornatissimus* | 30SWF39 | | DQ822171 | DQ822121 | MW817745 |
| IaAL05 | *Iberus gualterianus* | 30SWF08 | | AY928556 | AY928584 | - |
| Imari02 | *Iberus gualterianus mariae* | 30SWF26 | | DQ822170 | DQ822120 | - |
| Imari01 | *Iberus gualterianus mariae* | 30SWF26 | | DQ822169 | DQ822119 | - |
| IaAL04 | *Iberus gualterianus* | 30SWF07 | | AY928555 | AY928583 |  |
| IguaEHUMC2262 | *Iberus gualterianus mariae* | 36°51'00'' N -002°57'00'' E | | MW803159 | MW817705 | MW817744 |
| IgJ02 | *Iberus gualterianus gualterianus* | 30SVG28 | | AY928578 | AY928606 | - |
| IgJ01 | *Iberus gualterianus gualterianus* | 30SVG28 | | AY928577 | AY928605 | - |
| IgGR06 | *Iberus gualterianus gualterianus* | 30SVG32 | | AY928576 | AY928604 | - |
| IgAL03 | *Iberus gualterianus gualterianus* | 30SWF48 | | AY928570 | AY928598 | - |
| IgGR04 | *Iberus gualterianus gualterianus* | 30SVG32 | | AY928574 | AY928602 | - |
| IgGR03 | *Iberus gualterianus gualterianus* | 30SVG32 | | AY928573 | AY928601 | - |
| IgGR02 | *Iberus gualterianus gualterianus* | 30SVG32 | | AY928572 | AY928600 | - |
| IgGR01 | *Iberus gualterianus gualterianus* | 30SVG32 | | AY928571 | AY928599 | - |
| IgAL02 | *Iberus gualterianus gualterianus* | 30SWF48 | | AY928569 | AY928597 | - |
| IgAL01 | *Iberus gualterianus gualterianus* | 30SWF48 | | [AY928568](https://www.ncbi.nlm.nih.gov/nuccore/AY928568) | [AY928596](https://www.ncbi.nlm.nih.gov/nuccore/AY928596) | - |
| Ialo01A | *Iberus gualterianus alonensis* | 30SWF58 | | DQ822125 | DQ822075 | - |
| gua331 | *Iberus gualterianus* | Sierra Elvira | | KM592620 | KJ458530 | KJ458617 |
| guaESP331 | *Iberus gualterianus* | 37°24'56'' N 003º 43' 30"W | | KR705034 | KR704995 | KR705072 |
| IgJ03 | *Iberus gualterianus gualterianus* | 30SVG28 | | AY928579 | AY928607 | - |
| IgGR05 | *Iberus gualterianus gualterianus* | 30SVG32 | | AY928575 | AY928603 | - |
| Ilmin-150 (UPV) | *Iberellus minoricensis* | - | | DQ822174 | DQ822124 | - |
| MN 2527-Hel-161 | *Eremina dillwyniana* | - | | KR705036 | KR704999 | KR705076 |
| MN 2555-Hel-153 | *Rossmaessleria sicanoides* | - | | KR705040 | KR705003 | KR705080 |

**Table S3**

Morphometric parameters and ratios measured for *T. cilbanus* (*N* = 259).

| Parameters and ratios | Minimum | Maximum | Average | SD | CV (%) |
| --- | --- | --- | --- | --- | --- |
| Major Ø of the shell (mm) | 19.99 | 31.17 | 25.00 | 2.08 | 8.33 |
| Minor Ø of the shell (mm) | 16.95 | 26.24 | 21.05 | 1.80 | 8.56 |
| Shell height (mm) | 11.52 | 20.95 | 14.81 | 1.70 | 11.47 |
| Major external Ø of the peristome (mm) | 11.36 | 19.37 | 15.16 | 1.41 | 9.27 |
| Minor external Ø of the peristome (mm) | 9.61 | 15.88 | 12.34 | 1.13 | 9.17 |
| Shell height/Major Ø of the shell (ratio) | 0.49 | 0.73 | 0.59 | 0.04 | 6.54 |
| Shell area (mm^2^) | 267.69 | 642.38 | 416.13 | 69.36 | 16.67 |
| Peristome area (mm^2^) | 91.09 | 234.59 | 147.88 | 25.52 | 17.26 |
| Major Ø/Minor Ø of the shell (ratio) | 1.05 | 1.32 | 1.19 | 0.03 | 2.55 |
| Major Ø/Minor Ø of the peristome (ratio) | 0.99 | 1.51 | 1.23 | 0.08 | 6.53 |
| Peristome area x100/Shell area (%) | 28.80 | 45.08 | 35.61 | 2.84 | 7.99 |

**Table S4**

Morphometric comparisons between *T. cilbanus* and the two taxa of the *marmoratus* complex which inhabit the surrounding areas. K: Kruskal Wallis plus 2-tailed multiple comparison H test; A: one-way ANOVA plus post hoc Tukey test (HSD) for the comparisons between *T. cilbanus* and *I. marmoratus marmoratus* and *I. marmoratus loxanus,* respectively; ns: non-significant.

| Parameters and ratios | p-values | *T. cilbanus* comb. nov. (*N* = 259) | *I. marmoratus marmoratus* (*N* = 608) | *I. marmoratus  loxanus* (*N* = 808) |
| --- | --- | --- | --- | --- |
| Major Ø of the shell (mm) ^K, A^ | <0.001 | 25.00 ± 2.08 | 20.71 ± 1.83 | 22.42 ± 2.54 |
| Minor Ø of the shell (mm) ^K, A^ | <0.001 | 21.05 ± 1.80 | 17.43 ± 1.54 | 18.85 ± 2.16 |
| Shell height (mm) ^K, K^ | <0.001 | 14.81 ± 1.70 | 11.84 ± 1.54 | 11.59 ± 1.59 |
| Major external Ø of the peristome (mm) ^K, A^ | <0.001 | 15.16 ± 1.41 | 12.36 ± 4.26 | 13.39 ± 1.65 |
| Minor external Ø of the peristome (mm) ^K, A^ | <0.001 | 12.34 ± 1.13 | 10.07 ± 1.18 | 10.86 ± 1.37 |
| Shell height/Major Ø of the shell (ratio) ^K, K^ | <0.001 | 0.59 ± 0.04 | 0.57 ± 0.03 | 0.52 ± 0.03 |
| Shell area (mm^2^) ^K, K^ | <0.001 | 416.13 ± 69.36 | 283.68 ± 53.00 | 336.22 ± 77.57 |
| Peristome area (mm^2^) ^K, K^ | <0.001 | 147.88 ± 25.52 | 98.81 ± 39.66 | 115.82 ± 28.92 |
| Major Ø/Minor Ø of the shell (ratio) ^K, A^ | ns | 1.19 ± 0.03 | 1.20 ± 0.10 | 1.19 ± 0.03 |
| Major Ø/Minor Ø of the peristome (ratio) ^K, A^ | ns | 1.23 ± 0.08 | 1.23 ± 0.40 | 1.24 ± 0.08 |
| Peristome area x100/Shell area (%) ^K, A^ | <0.001 | 35.61 ± 2.84 | 34.85 ± 12.23 | 34.47 ± 3.05 |
